# Perceptions of home hospitalization among the public and physicians in Israel: findings from surveys conducted for the Dead Sea Health Policy Conference of 2022

**DOI:** 10.1186/s13584-024-00654-4

**Published:** 2024-11-29

**Authors:** Michal Laron, Rachel Nissanholtz-Gannot, Sharvit Fialco, Inbal Halevi Hochwald, Gizell Green, Itamar Offer, Gil Lavie

**Affiliations:** 1 Health Policy Team, The Myers-JDC-Brookdale Institute JDC Hill, POB 3886, Jerusalem, 9103702 Israel; 2 School of Health Sciences, Ariel University, Ariel, Israel; 3 Nursing Department, Max Stern Yezreel Valley College, The Yezreel Valley, Jezreel Valley, Israel; 4 Department of Nursing, The Max Stern Yezreel Valley College, Israel Senior Research Coordinator, Shoham Geriatric Center, Ministry of Health, Yezreel Valley , Israel; 5 Formerly Sabar Health – Hospital at Home, Netanya, Israel; 6 Branch of Planning and Strategy, Clalit Health Services, Tel Aviv, Israel; 7 Ruth and Bruce Rappaport Faculty of Medicine, Israel Institute of Technology, Technion, Haifa, Israel

**Keywords:** Hospital at home, Patient-centered care, Dead Sea Conference on Health Policy

## Abstract

**Background:**

Hospital at Home (HaH) is an alternative care model that provides acute hospital-level services to patients at their homes. Despite its proven advantages and global experience, HaH did not gain significant traction in Israel until the COVID-19 pandemic. The issue was highlighted at the 2022 Dead Sea Conference on Health Policy. This study compares perceptions of HaH among the Israeli public and physicians, Jewish and Arab, identifying facilitators and barriers to its expansion in Israel.

**Methods:**

Two online surveys were conducted, one with 342 physicians and another with 424 members of the public aged 35+. Respondents were sampled based on age, gender, district of residence, and population group. Descriptive statistics and chi-square tests explored perceptions, and logistic regression analyzed multivariate relationships.

**Results:**

Results showed 39% of the public believed HaH care quality is as good as or better than hospitals, compared to 65% of physicians. 44% of the public felt HaH safety is as good or better, while 75% of physicians agreed. 58% of the public saw communication between patients/families and the healthcare professionals in HaH as good or better, contrasted with 91% of physicians. 78% of the public and 97% of physicians viewed HaH as a good alternative to hospitalization and would consider using it personally. Arab and lower-income respondents were less positive about HaH than Jewish and higher-income respondents. Community-based physicians preferred HaH more than hospital-based ones. Barriers to HaH expansion included lack of specialized manpower, resources, and awareness.

**Conclusions:**

The findings suggest that both the public and physicians show confidence in HaH, and it is gaining popularity among both. Policymakers could use these insights to expand HaH, focusing on increasing awareness, reducing family burden, tailoring services for diverse populations, involving hospital staff, and investing in resources and training.

## Background

The provision of care in a patient’s home in a variety of clinical situations and for various degrees of acuteness has existed in Israel for several decades. The home-care units were established already in the 1970s and since then the service has expanded in all the Health Maintenance Organizations (hereafter: HMOs) to include medical, rehabilitative, and nursing care. Acute medical home care, also known as ”Hospital at Home” (hereafter: HaH), is a service provided in the patient’s home as an alternative to hospitalization in an internal medicine ward, involving intensive medical support and care delivered by multidisciplinary staff.

The expansion of HaH is a response to the increase in the demand for medical services due to demographic and chronic morbidity trends, the shortage of hospital beds, the expense of in-patient care, and greater awareness of the risk attached to a hospital stay, in particular of iatrogenic complications resulting from a long hospitalization [1]. Studies in other countries (primarily in the US [2, 3] and Australia [4]) have demonstrated that HaH can be provided safely and effectively, with minimal iatrogenic complications, can earn high satisfaction ratings among patients, and that the costs of care are reduced in HaH. Despite the rationale underlying HaH and the experience with HaH that has accumulated worldwide, it did not develop to a significant extent in Israel until 2020, during the COVID-19 pandemic, which served as a “stress test” for the ability of the health system to provide a rapid and large-scale response to many patients in need of hospitalization. Following the demonstration of its feasibility during the pandemic, alongside technological innovations such as those which facilitate remote care, and patient preference for home care, the conditions became ripe to expand HaH services in Israel.

The Dead Sea Conference is an annual health policy gathering organized by the Israel National Institute for Health Policy Research to promote discussion of health policy issues and improve Israel’s healthcare system. The conference that took place in December 2022 focused on challenges implicit in hospital-community relations and provision of HaH services. In preparation for the conference, our research team conducted surveys of diverse population sectors in Israel with the support of the National Institute for Health Policy Research. The goals of the surveys were to determine perceptions of HaH among the public (potential patients and their families), and among physicians, to identify facilitators and barriers to expansion of HaH services. This article presents the main findings of the surveys and their implications for the future of HaH.

To the best of our knowledge, no previous large-scale surveys of stakeholders’ perceptions of HaH have been conducted in Israel. Marziano et al. [5] have retrospectively described and analyzed data collected for patients admitted to the Sheba beyond’s HAH services. Punchik et al. [6] have characterized the service model of Home Care Unit for homebound patients 65 years of age and above. Studies in other countries have mostly focused only on patients and their families. Only a few studies have looked at physicians’ attitudes toward HaH, and even fewer have compared the attitudes of physicians to those of patients and families, which is the focus of this article.

## Methods

### Study sample and data collection

Two online cross-sectional surveys were conducted, one for physicians, and the other for the public. The survey among the public was based on a sample of Israelis, aged over 35 (since HaH is usually not common among younger age groups). The survey was conducted during November 6–13, 2022, through an online panel by the Tovanot Company. The survey questionnaire was available in Hebrew and Arabic. Quota sampling was used to ensure adequate representation of four quota variables, age; gender; district of residence; population group (Jewish or Arab). The online questionnaire was sent to 3,294 Jewish panelists (response rate 40%) and to 1,162 Arab panelists (response rate 27%) (because we used quota sampling, not all individuals who were approached by the panel company and responded were ultimately included in the study). All quotas were reached^1^.

The physicians’ sample was based on a random sample of primary care and internal medicine physicians, irrespective of whether they practiced full- or part-time, and whether they practiced in a hospital or community setting. The digital questionnaire was distributed by an email from the Association of Family Medicine and the Association of Internal Medicine to all their members, November 13–26, 2022.

The preparation of this survey and the analysis of the responses were conducted by the Myers-JDC-Brookdale Institute.

## The questionnaires

The questionnaire for the public was designed to elicit perceptions of HaH vs. hospital care concerning various aspects of the healthcare experience, including the quality and safety of treatment, communication between patients/families and healthcare professionals, the caregiving burden on the patient’s family, and whether HaH is a preferable solution over traditional hospitalization. In addition to the closed-ended questions, the survey included an open-ended question concerning the perceived advantages of HaH in comparison to hospitalization.

The survey of physicians used similar parameters to make the comparison and was intended to highlight any differences in perceptions between physicians and the public. Furthermore, the physicians were given an open-ended question about perceived barriers to the expansion of HaH services.

The survey of both groups was conducted concurrently.

### Data analysis

The data was analyzed using IBM Statistical Package for the Social Sciences (SPSS) No. 28. Non-responses to the close-ended questions were treated as missing values. We used descriptive statistics and frequencies to examine the perceptions of HaH among the physicians and among the public. The degree of association between the variables, measured on a nominal scale, was examined using the chi-square test. Thereafter, the responses were further differentiated according to the main workplace of the physician (community or hospital), and by population group (Jewish or Arab) and income of the public. A value of *p* < 0.05 was considered significant. Bivariate relationships were analyzed using the χ2 test, and multivariate relationships were analyzed using logistic regression.

## Ethics

Since the study questionnaires did not include items involving specific patients and/or personally identifiable information, the legal advisor of the Myers-JDC-Brookdale Institute exempted this study from its IRB procedure. The introductory text of the questionnaire explained the objectives of the study and assured potential participants of anonymity. All participants gave written informed consent to participate.

## Results

In the survey of the public, there were 424 respondents above the age of 35, average age of 51, 81% were Jews, 51% were women. See Table 1. The response rate was 40% among Jews and 27% among Arabs.

In the survey of physicians, there were 342 respondents, average age of 52, 93% were Jews, 49% were women. Those who practiced mainly in community settings constituted 71%, and 29% practiced in hospitals. Except for 2% of the physicians who did not claim to be specialists, the doctors practiced in the specialties of family medicine (62%), internal medicine (33%) and pediatrics or another (3%), with 76% having practiced medicine for over 11 years, and 41% were in managerial positions at the time of the survey. In the year before completing the questionnaire, 73% had referred patients to HaH, and 11% of the physicians had personal experience with HaH (either they or a member of their family or a close friend had been treated in a HaH setting). See Table 2.

The sample of the public is representative of four variables: age, district of residence, population group, and gender, with over-representation of individuals with academic education. The sample of the physicians is representative according to gender, district of residence, and age distribution, with under-representation of Arab physicians - for more see the section of study limitations.

**Table 1:** Respondents characteristics – the public (*N* = 424)

Background Characteristic		%
Gender	Female	51
	Male	49
Population group	Jewish	81
	Arab	19
Age	35–49	51
	50–69	45
	70+	4
Income	Well above average	9
	Slightly above average	21
	Average	22
	Slightly below average	17
	Well below average	25
	N/A	6
Education	Non-academic	40
	Academic	60
District of residence	Center and Tel Aviv	45
	North	19
	Haifa	14
	Jerusalem and the West Bank	10
	South	12
He or a person close to him has received HaH care in the last two years	Yes	12
	No	88
He or a person close to him was hospitalized in the internal medicine ward in the last two years	Yes	33
	No	67

**Table 2:** Respondents characteristics – physicians (*N* = 342)

Background Characteristic		%
Gender	Female	51
	Male	49
Population group	Jewish	93
	Arab	7
Age	<=35	8
	36–50	42
	51+	50
Main place of practice	Hospital	29
	Community	71
Specialty	Family medicine	62
	Internal medicine	33
	Pediatric or another from the two above	3
	No specialty	2
Years since graduation from medical school	≤ 5	9
	6–10	15
	11–15	12
	+ 16	64
Works in a managerial position	Yes	41
	No	59
District of practice	Center and Tel Aviv	49
	North	16
	Haifa	12
	Jerusalem and the West Bank	13
	South	10
In the last year, physician referred a patient to HaH	Yes	73
	No	27
In the last year, physician or relative or friend was treated via HaH	Yes	11
	No	89

A comparison of the various perceptions of treatment quality, safety, and communication between the physicians and patient/family respondents revealed that the physicians had greater confidence in HaH than did the general public respondents. See Table 3. Among the public respondents, 61% felt that quality of care is better in a hospital than at home, as compared to only 36% of the physicians. Only 39% of the public respondents felt that the quality of care provided in HaH is similar (21%) or better (18%) than at a hospital, as compared to 64% of the physicians (47% of them felt the quality of care provided in HaH is similar and 17% of the physicians felt it is better) (χ²=50.67, *p* < 0.000).

Concerning treatment safety, the most common response among physicians (44%) was that home hospitalization is safer than HaH, as compared to 29% of the public respondents. In contrast, 56% of the public respondents believed that care at a hospital is safer than at home, as compared to 25% of the physicians (χ²=67.65, *p* < 0.000).

A high majority of the physicians (76%) believed that communication between the physicians and patient/family is better in a HaH setting than at a hospital, as compared to 45% of the public respondents, 42% of whom felt that communication is better in a hospital setting as compared to only 9% of the physicians (χ²=98.52, *p* < 0.000). See Table 3.

**Table 3:** Comparison of perceptions of HaH and hospitalization treatment quality, safety, and communication

Questions	Answers	Physicians (%) (*N* = 342)	Public (%) (*N* = 424)	Sig (*P*)	χ²
The quality and professionalism of the treatment provided is	Better in HaH	17	18	0.000	50.67
	Better in the hospital	36	61		
	Similar in both	47	21		
The safety of treatment is	Better in HaH	44	29	0.000	67.65
	Better in the hospital	25	56		
	Similar in both	31	15		
The communication between patient/family and physicians is	Better in HaH	76	45	0.000	98.52
	Better in the hospital	9	42		
	Similar in both	15	13		

**Fig. 1:**
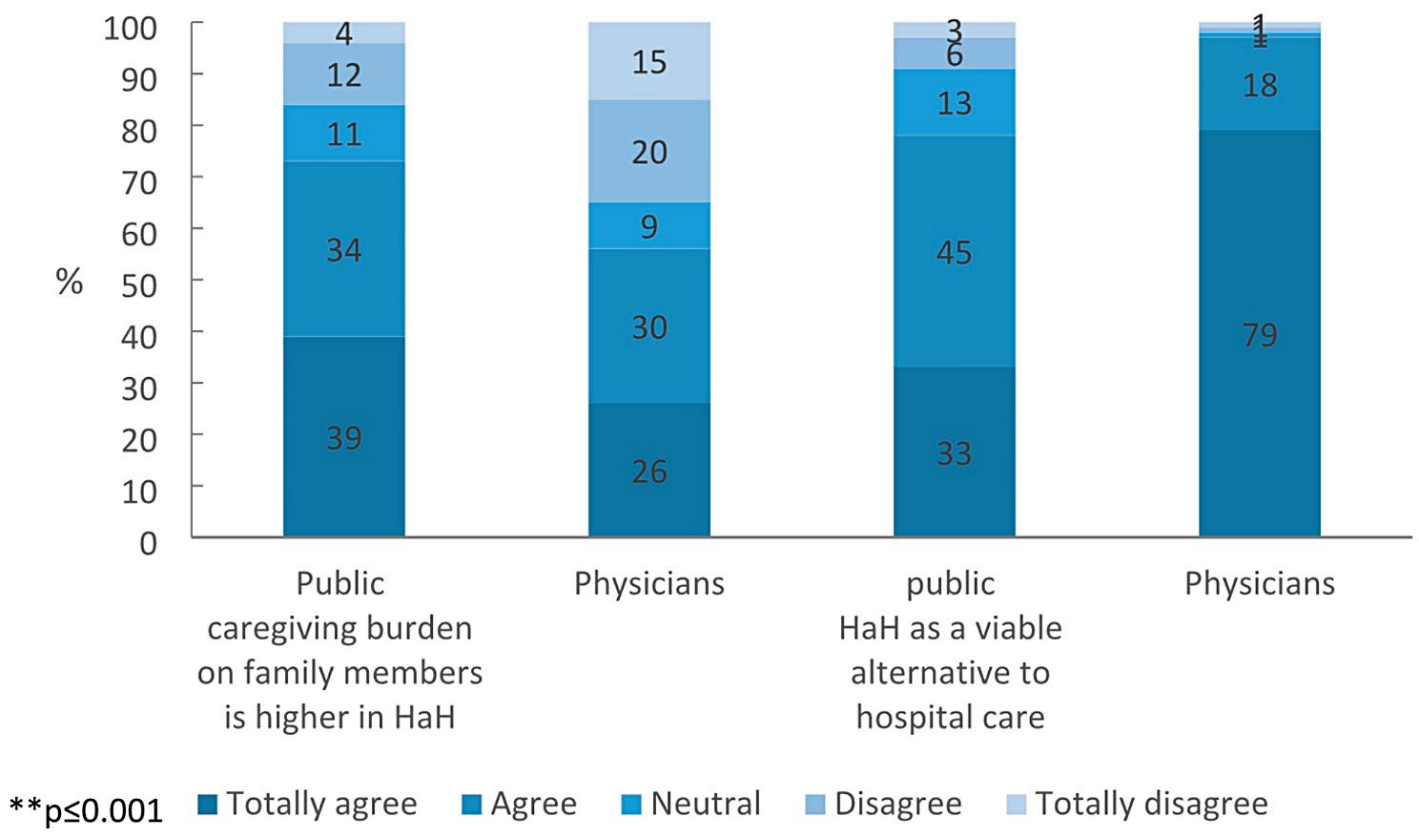
Comparison of the public and physicians’ attitudes toward home hospitalization (%)

Concerning the caregiving burden on family members, 73% of the public respondents totally agreed (39%) or agreed (34%) that it is higher in HaH than in a hospital, compared to 56% of the physicians (26% totally agreed and 30% agreed) (χ²=44.67, *p* < 0.000). A high majority of the public respondents (78%) agreed, either moderately (45%) or strongly (33%), with the statement, “Home hospitalization is a viable alternative to hospital care for the appropriate patient group,” as compared to nearly all (97%) the physicians (18% moderately and 79% strongly agreed) (χ²=166.55, *p* < 000). See Fig. 1.

**Fig. 2:**
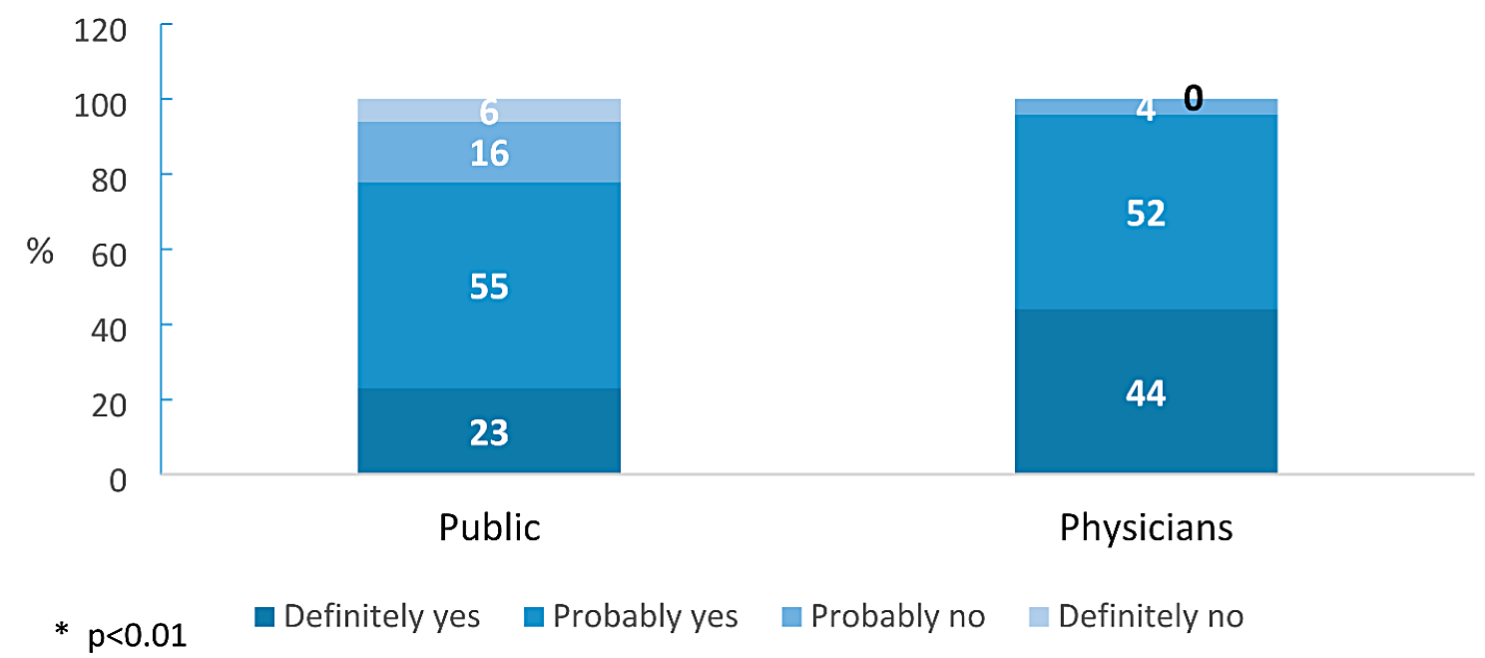
Willingness to consider HaH instead of hospital admission for needed medical treatment (%)

Very high percentages of the public respondents (78%) and physicians (96%) indicated their willingness to consider HaH if offered. However, a substantial divergence emerged between the two groups regarding the degree of their preference. Specifically, the proportion of physicians who expressed a strong preference for HaH was twice that of the public respondents (44% vs. 23%). In contrast, the proportion of the public respondents (22%) who indicated a lack of interest in home hospitalization (16% probably and 6% definitely) was more than fivefold that of physicians (4% probably and 0% definitely) (χ²=68.01, *p* < 000). See Fig. 2.

### The public perceptions according to population group (jewish or arab) and other background characteristics

**Fig. 3:**
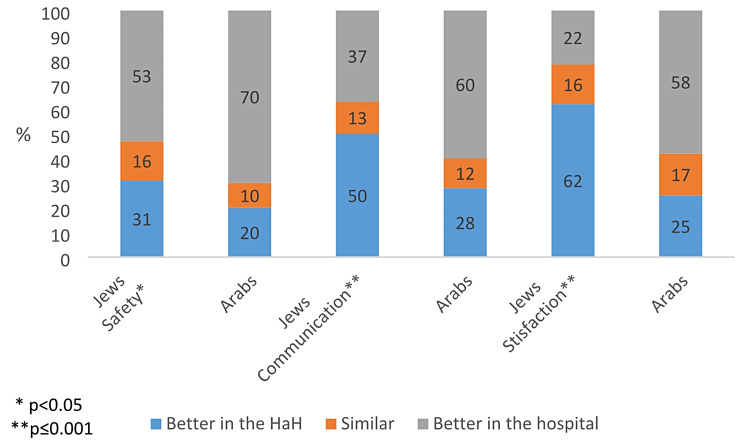
Attitudes towards HaH versus hospitalization by population group (Jewish or Arab) (%)

Across most questions, a lower proportion of Arab respondents had a positive view of HaH relative to Jewish respondents. Only 20% of Arabs considered HaH to be safer than hospitalization, compared to 31% of Jews (χ²=7.06, *p* = 0.03), and 28% of Arab respondents believed that communication between the patient/family and healthcare professionals is better in HaH, compared to 50% of Jews (χ²=14.16, *p* = 0.001). Only 25% of Arab respondents believed that patient satisfaction is higher with HaH than with hospitalization, while 62% of Jews believed so (χ²=40.09, *p* < 0.00). See Fig. 3. These differences were significant even in a multivariate logistic regression analysis, where the variables controlled were income level, gender, age, and district of residence (not shown in this article).

In response to the question, “If you or a family member needed medical treatment, would you be interested in home hospitalization rather than hospitalization in a hospital setting?”, 81% of Jewish respondents said they were likely or very likely to choose HaH, as compared to 67% of Arab respondents (χ²=5.92, *p* = 0.015) (OR = 2.1, *p* = 0.01). See Table 4, Model A. The difference between Jewish and Arab respondents became non-significant in multivariate logistic regression analysis when the variable of income was added to the regression, such that 83% of respondents with similar or higher-than-average income favored HaH in case of need, compared to 72% of those whose income was below-average (χ²=5.58, *p* = 0.01) (OR = 1.8, *p* = 0.03). There were no significant differences in perception regarding safety, quality of care, and quality of communication according to income level. Nor were significant differences found in perceptions regarding HaH according to gender, age group, education, and district of residence in bivariate and multivariate analysis. See Table 4 Model B.

**Table 4:** Odds ratios for respondents interested in HaH if medical treatment was needed

	Model A			Model B Income variable added to the regression		
Predictor	Odds Ratio	95% CI	*P*	Odds Ratio	95% CI	*P*
Gender (Men)	1.328	(0.799–2.206)	0.274	1.374	(0.810–2.330)	0.239
Population group (Jewish)	2.136	(1.162–3.925)	0.014	1.625	(0.850–3.108)	0.142
Income (Average and above)	---	---	---	1.839	(1.061–3.187)	0.03
Age group (35–49)	0.88	(0.531–1.458)	0.619	0.771	(0.457–1.302)	0.331
District (Center)	0.846	(0.482–1.485)	0.56	0.763	(0.429–1.359)	0.359
Constant	1.538		0.400	1.533		0.436

### Physicians’ perceptions according to whether they practiced in a hospital or community setting

**Fig. 4:**
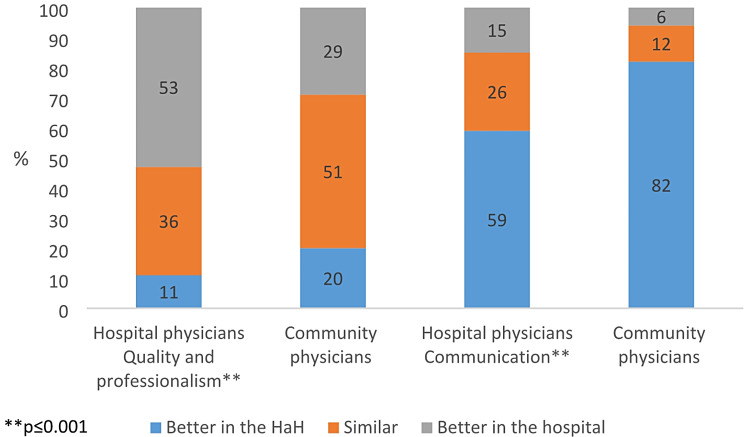
Physician attitudes regarding HaH versus hospitalization according to main workplace (%)

Physicians who practiced mainly in a community setting (“community physicians”) indicated a stronger preference for HaH over hospitalization than did physicians who mainly practiced in a hospital (“hospital physicians”). Among community physicians, 71% believed that the quality of treatment at home is either similar (51%) or better (20%) than treatment in a hospital. Among hospital physicians, 47% believed that the quality of treatment at home is either similar (36%) or better (11%) than treatment in a hospital (χ²=13.06, *p* = 0.001). About 82% of the community physicians believed that communication between patient/families and healthcare professionals is better in HaH than in hospitals. Although a smaller proportion of hospital physicians (59%) shared this opinion, they still represent a majority of that group (χ²=15.90, *p* = 0.000). See Fig. 4.

### Open-ended questions

The questionnaire for the public respondents included an open-ended question concerning the perceived advantages of HaH in comparison to hospitalization. Among the public respondents, 60% indicated that HaH was preferable because it is convenient, familiar, private, tranquil, and comfortable. Additional benefits noted were a lower risk of infection (40%) and easier access for family members to provide patient support while maintaining their daily routines (23%). Among this group, 12% felt that HaH allowed for more personal attention from healthcare professionals and involved less bureaucratic obstacles than hospitalization.

**Fig. 5:**
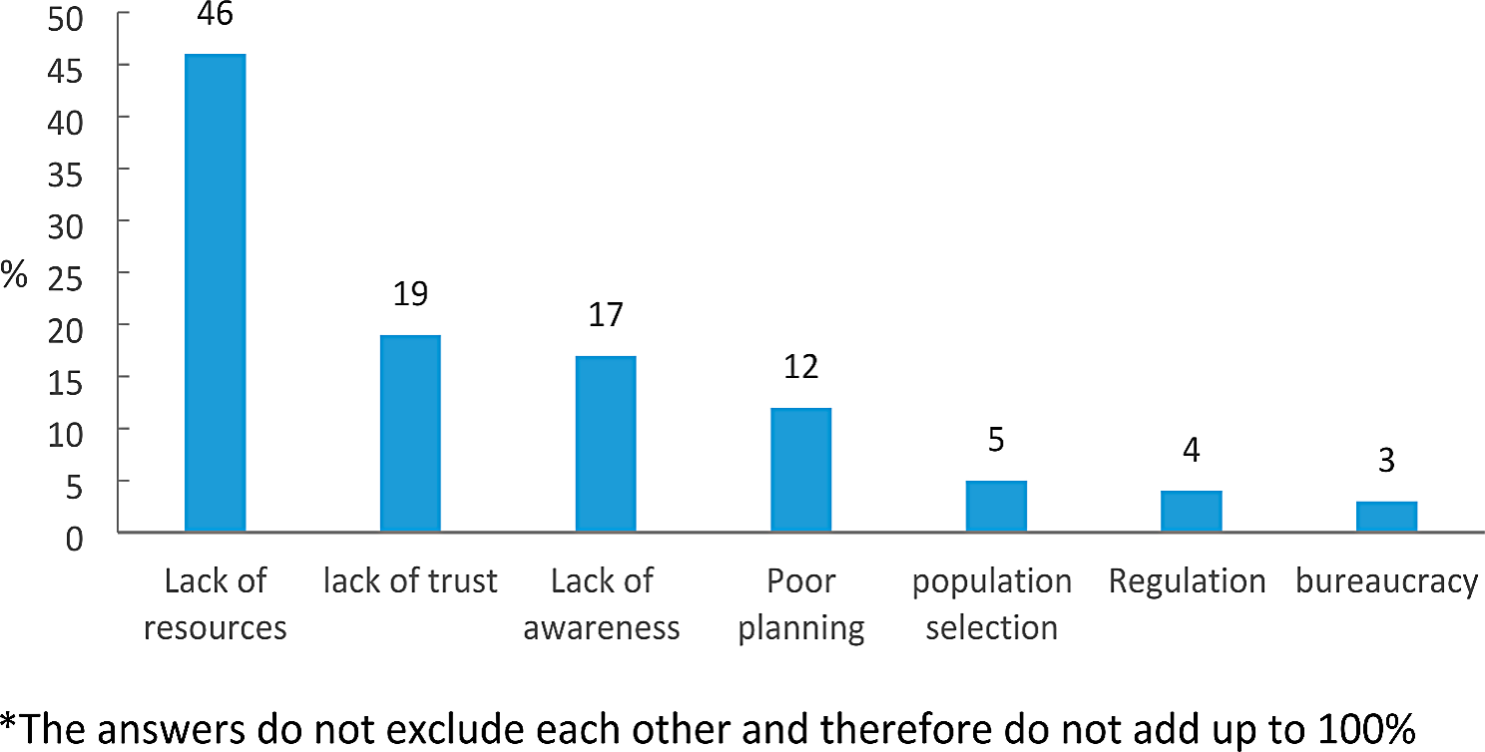
Current Barriers to HaH Expansion in Israel: Physicians’ Perspectives by Category, %*

The open-ended question in the physicians’ questionnaire concerned perceived barriers to the expansion of HaH services. As illustrated in Fig. 5, three themes emerged. Close to half (46%) of the physicians expressed the opinion that due to a lack of human and financial capital, Israel lacked enough qualified physicians and nurses to adequately provide HaH services. About a fifth of them (19%) thought patients and their families lack trust in the viability of HaH, and hospitals lacked motivation to admit patients to HaH care. A slightly smaller number (17%) perceived that patients, families, and physicians were not aware of the availability of the HaH option.

## Discussion

The results indicate that a large majority (78%) of the public and nearly all the physicians (97%) viewed HaH as a good alternative to hospitalization and these proportions were similar in the respective groups’ results concerning interest in using HAH personally (for them or for a family member) if offered.

However, while both the public respondents and physicians perceived the HaH healthcare model favorably, with potential for providing high-quality and safe medical services, the proportion of the public respondents with this view was lower than that of the physicians.

While there have not been any previous large-scale surveys of stakeholders’ perceptions of HaH in Israel, various studies worldwide have focused on patients and their families. Our findings concerning the attitudes of the public are in line with those of studies conducted in other countries, which also found that patients viewed HaH favorably, and felt it to be efficient, safe, and patient-centered [7]. Two recent interview-based studies in Singapore examined the perceptions and experiences of patients and their caregivers concerning HaH care. Their findings, like similar studies in the USA [8] and the UK [9], suggested that the respondents associated the patient-centeredness of HaH with comfort, convenience, and higher quality of care [10], and that HaH was perceived as an appropriate alternative for managing high-risk patients at home [11]. A 2019 qualitative study in France [12] found that patients and family caregivers associated HaH with higher quality of care as well as increased morale.

A qualitative study in 2020 in Singapore involving patients and physicians found that most stakeholders embraced HaH [13]. A 2021 survey article [14] brought together the findings of qualitative studies that examined the perceptions of HaH among both patients and healthcare professionals, and reported that both groups noted home environment, proximity to family members, individualized treatment, and the perception of improved clinical outcomes as significant advantages of HaH. Patients reported experiencing improved sleep, better appetite, and a speedier recovery. Health care professionals observed that the familiarity and privacy of home encouraged patients to “reveal something they would never mention while in the hospital,” thus facilitating better care. However, this survey of quantitative study examined the perceptions of patients being treated and physicians participating in this service, reporting on their personal experiences, while this current study refers to the general public’s perceptions and physicians, whether they experienced the service or not. Also, the quantitative studies that were mentioned did not offer comparisons between the perceptions of patients and healthcare professionals.

As in our study, which showed that the public respondents (58%) and the physicians (91%) expressed high regard for HaH concerning communication between the patient/family and the healthcare professionals, the survey of qualitative studies cited above [14] reported that healthcare professionals commented that they had more opportunities to collaborate with their patients in HaH than in a hospital setting. In HaH healthcare professionals felt they were not merely regarded as medical staff, but more like guests invited into patients’ homes. Since healthcare professionals were able to assess patients in their natural home environment, both parties highlighted that medical advice was more fine-tuned to specific situations in their everyday lives.

Regarding the quality, safety and professionalism of the treatment rendered, most of the public respondents expressed greater confidence in the quality of the care available at a hospital (61%) and its safety (56%) than in HaH. Higher rates of confidence in HaH quality and safety among physicians may be attributable to physicians’ closer familiarity with the respective types of services available at hospitals and in HaH. In fact, 73% of the physicians had referred a patient to HaH during the year preceding the survey, while only a minority of the public respondents experienced the rendering of HaH services. Lack of confidence in HaH and lack of awareness of its availability were mentioned by physicians as the second and third most common obstacles to the expansion of HaH in Israel. Low public awareness of the availability of HaH has also been observed in studies in other countries [15]. Thus, a key challenge for the healthcare community and policy makers is to raise public awareness of the availability of HaH.

Both the public respondents and physicians felt that caregiver burden imposed on family is greater in HaH than in hospitalization, with a higher proportion of the public respondents (73%) expressing this opinion than physicians (56%). Other studies have reported the greater role of caregivers in HaH than in hospitalization [12, 14, 16]. This perception would seem to be another barrier to the expansion of HaH. Therefore, the healthcare community and policy makers should consider the extent to which HaH services ought to include professional caregiving services, such as long-term nursing assistance, to augment the informal caregiving services provided by family members in order to lessen family caregiver burden and the risk of caregiver burnout. In some cases, family members may simply not be able to serve as caregivers or may be more susceptible to burnout than average. Such scenarios may render HaH unfeasible for some patients, and pre-admission assessments should carefully screen for possibilities of this nature.

The segmentation of the questionnaire responses enabled us to find differences in perception between various groups. Arab respondents expressed a less positive attitude toward HaH vs. hospitalization than did Jewish respondents. Although most Arab respondents viewed HaH as a viable alternative to hospitalization and were interested in using it, a higher proportion of them than Jewish respondents felt that the level of safety is significantly higher in a hospital than in HaH, irrespective of economic status. This finding may be related to historically lower levels of utilization and familiarity with HaH among Arabs [17]. Studies in other countries have found a relationship between cultural and social attitudes among minority populations and their healthcare preferences [18, 19]. Research in Israel has revealed that when emergency medical care is needed, Arabs turn to hospital emergency rooms at a higher rate than do Jews, who utilize community emergency centers at a higher rate [17]. More research on the geographic distribution of health services and into cultural and social factors affecting healthcare choices is warranted to facilitate greater utilization of HaH among Arabs.

Income level also was found not to be related to the respondents’ perceptions regarding HAH quality and communication, but a smaller proportion of low-income individuals were willing to be hospitalized at home compared to other income groups. This result may reflect less than optimal conditions for care within lower income patients’ homes. Thus, the quality of home conditions should also be assessed as part of the HaH pre-admission process.

This and more, our logistic regression analysis showed that when the income variable was added to the regression model regarding willingness to utilize HaH, the previously significant association between the population group (Arabs) and willingness to utilize HaH in Table 4 Model A became non-significant in Model B, while the correlation between income and willingness to use HaH remained significant. Thus, the difference we found between Arabs and Jews regarding the willingness to utilize HaH may stem not only from lower historical utilization rates, lesser familiarity with HaH, and cultural/social attitudes among the Arab population but from the higher percentage of Arabs with below-average income in the population^2^.

Perhaps not surprisingly, physicians who practiced mainly in hospitals expressed lower regard for HaH than physicians who practiced in the community. The division of roles in Israel between the community and the hospital concerning HaH is unique. Thus, while in most countries that have taken the lead in adopting HaH provision (such as Spain, the US, and Australia) the hospitals play a leading role in HaH through community outreach, in Israel it is the country’s four HMOs that serve this function. The configuration of this activity is based on the directives of the Ministry of Health regarding the responsibility of the HMOs to provide HaH services [20]. Most HaH patients in Israel are admitted to HaH via the HMOs before even arriving at the hospital (the emergency room or hospitalization in a ward). Only a small proportion of patients are referred to HaH after receiving care in a hospital and an even smaller proportion are treated by medical staff working in the hospitals.

In addition to the aforementioned directives and work processes, in their preparation for Israel’s 2022 Dead Sea Conference, the authors mapped the barriers to expansion of HaH, and found conflicts of interests between hospitals and community districts regarding maintaining and expanding HaH activity, including conflicting financial incentives related to a possible reduction in the hospitals’ scope of activity; concern about change in the patient population in hospitals and increased share of acute patients in the hospitals; the undermining of hospitals’ long-term development plans; and lack of acquaintance and trust between hospital and HMO staff regarding optimal patient placement [21].

This may explain the lower level of support for HaH among hospital physician’s vs. community physicians. In order to facilitate the expansion of HaH in Israel and its establishment as a central component of the health system These conflicts should be addressed through recruiting the support of hospital staffs, including the alignment of incentives; improving familiarity with HaH activity and with the abilities of the HMOs medical system; and promotion of their partnership and active involvement in the design and provision of HaH services.

The findings of our survey of physicians also revealed some major barriers that may be hindering the expansion of HaH in Israel, including the lack of specialized manpower needed to expand the services; the lack of budget resources; a lack of awareness that the service exists; and a lack of interest among stakeholders to expand a new service or a conservative attitude toward hospitalization. These findings can provide policymakers with insights that can help to eliminate barriers and facilitate a significant expansion of HaH services.

### Study limitations

By design, our sample of the public is representative of four variables: age, district of residence, population group, and gender. Nevertheless, responses may be subject to bias because of the over-representation of individuals with academic education – 60% compared to 50% in the general population.^3^The attributes of the responding Family and Internal physicians we recruited were equated with those of specialists in internal medicine and family medicine based on data from the Ministry of Health.^4^ We found that the physician sample was representative according to gender, (49% were men, compared to 55% in Israel’s physician population) district of residence (49% were from the center of Israel, compared to 50% in Israel’ physician population), and age distribution (which was similar for the sample and the population), but our study was under-representative of Arab physicians in Israel (7% compared to 23% in the population).^3^ Efforts should be made to elicit more input from Arab physicians, especially given our findings concerning less positive attitude toward HaH in the Arab sector.

## Conclusions

To the best of our knowledge, this is the first study in Israel to examine the perceptions of HaH by patient/family and physician stakeholders. The main strength and value of this research is its contribution to the understanding of factors among both consumers and providers of healthcare services that may suggest initiatives that can be undertaken to strengthen and expand HaH. As such, the study provides the healthcare community and policy makers with important data and insights concerning the future of acute care in Israel. The perceptions of the public respondents and physicians suggest there is considerable sentiment that HaH is a viable mode of providing acute care, and that there is substantial support for strengthening and expanding HaH. Accordingly, we offer the following recommendations:

1. Increasing awareness – Efforts should be invested to boost awareness of HaH among physicians and patients/families, especially among Arabs.
2. Reducing the burden on family caregivers – Policy and practice development should consider the issue of HaH caregiver burden and the feasibility and cost effectiveness of adding a long-term nursing assistance component to HaH in certain cases.
3. Differentiation according to population group – Further research should be conducted to tailor HaH to the various sectors of the Israeli population, with the goal of expanding HaH in an equitable and culturally sensitive manner.
4. Introducing sophisticated pre-admission assessment tools – Tools should be developed and refined to screen patients, such as for whether family members of patients are able to server as caregivers during HaH and whether patient living conditions are suitable for in-home treatment.
5. Involvement of hospital staffs – Hospital staffs should be recruited to the effort, including the alignment of incentives, the improvement of their familiarity with HaH services and the abilities of the community medical system, and their involvement in the design and provision of HaH services.
6. Resources – Greater financial resources should be made available for expanding HaH, including the training of larger numbers of specialized healthcare professionals needed for this expansion.

## Data Availability

The dataset used and analyzed during the current study is available from the corresponding author upon reasonable request.

## Footnotes

1 According to the social survey of the Central Bureau of Statistics for 2022.

2 The social survey of the Central Bureau of Statistics for 2022.

3 OECD (2022), Education at a Glance Database, http://stats.oecd.org/.

4 According to the data of the POWERBI report of the Ministry of Health, Division of Strategic and Economic Planning, 23.12.23.
